# The Economic Impact of Malignant Catarrhal Fever on Pastoralist Livelihoods

**DOI:** 10.1371/journal.pone.0116059

**Published:** 2015-01-28

**Authors:** Felix Lankester, Ahmed Lugelo, Rudovick Kazwala, Julius Keyyu, Sarah Cleaveland, Jonathan Yoder

**Affiliations:** 1 Paul G. Allen School for Global Animal Health, Washington State University, Pullman, Washington, United States of America; 2 Institute of Biodiversity, Animal Health and Comparative Medicine, University of Glasgow, Glasgow, United Kingdom; 3 Faculty of Veterinary Medicine, Sokoine University of Agriculture, Morogoro, Tanzania; 4 Tanzanian Wildlife Research Institute, Arusha, Tanzania; 5 School of Economics, Washington State University, Pullman, Washington, United States of America; University of Liverpool, UNITED KINGDOM

## Abstract

This study is the first to partially quantify the potential economic benefits that a vaccine, effective at protecting cattle against malignant catarrhal fever (MCF), could accrue to pastoralists living in East Africa. The benefits would result from the removal of household resource and management costs that are traditionally incurred avoiding the disease. MCF, a fatal disease of cattle caused by a virus transmitted from wildebeest calves, has plagued Maasai communities in East Africa for generations. The threat of the disease forces the Maasai to move cattle to less productive grazing areas to avoid wildebeest during calving season when forage quality is critical. To assess the management and resource costs associated with moving, we used household survey data. To estimate the costs associated with changes in livestock body condition that result from being herded away from wildebeest calving grounds, we exploited an ongoing MCF vaccine field trial and we used a hedonic price regression, a statistical model that allows estimation of the marginal contribution of a good’s attributes to its market price. We found that 90 percent of households move, on average, 82 percent of all cattle away from home to avoid MCF. In doing so, a herd’s productive contributions to the household was reduced, with 64 percent of milk being unavailable for sale or consumption by the family members remaining at the boma (the children, women, and the elderly). In contrast cattle that remained on the wildebeest calving grounds during the calving season (and survived MCF) remained fully productive to the family and gained body condition compared to cattle that moved away. This gain was, however, short-lived. We estimated the market value of these condition gains and losses using hedonic regression. The value of a vaccine for MCF is the removal of the costs incurred in avoiding the disease.

## Introduction

**Figure 1:**
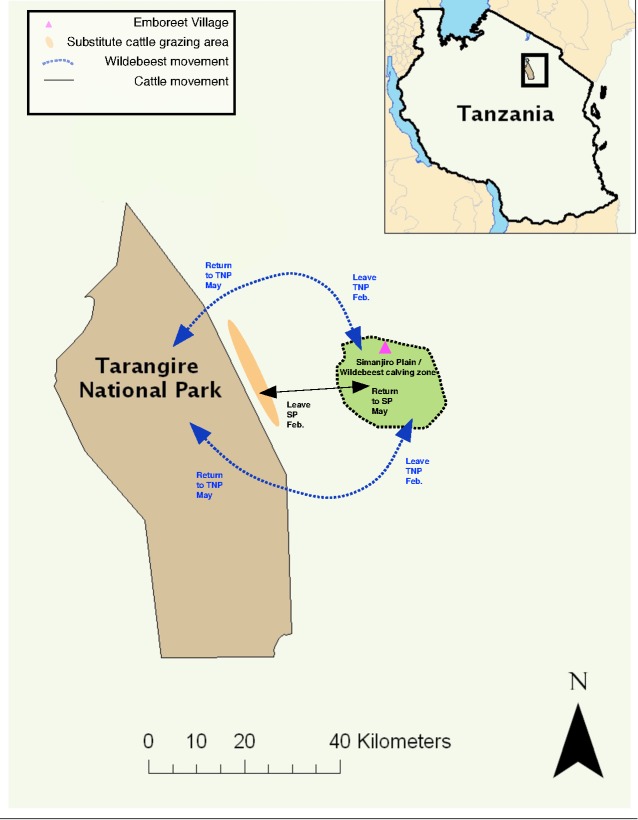
A map of the study site. Indicated are the location of Tarangire National Park (TNP), the Simanjiro Plain (SP), Emboreet Village (pink triangle), the wildebeest migration routes (blue broken line) and the direction the cattle travel (black solid line) to find substitute grazing pastures (orange area). Source: Map created by Thomas Morrison and Felix Lankester

Wildebeest-associated malignant catarrhal fever (MCF) is an often-lethal disease that affects cattle and is a particular problem for pastoralists living in eastern and southern Africa [1, 2]. The disease is caused by a gamma herpes virus, Alcelaphine herpesvirus I (AlHV-1), which is excreted by wildebeest calves (*Connochaetes taurinus*) in the three months following the brief annual calving period [3]. Consequently being in proximity to wildebeest is only a risk to cattle for a specific and limited period each year. Although sedentary populations do exist, the wildebeest is predominantly considered a migratory herbivore that specialises on feeding on short, green grass and, in many populations, it returns annually to the same pastures to give birth and suckle its young, with the timing of its arrival being linked with seasonal rainfall [4]. If located outside of a national park these ancestral calving ground pastures may also be utilized by domestic livestock in mixed-use buffer zones (Fig. 1). Because wildebeest calves pose a risk of MCF, livestock owners living in these zones face a dilemma: to move their cattle away from the wildebeest calving grounds on to more marginal land (substitute pastures) at a time of year when the new pasture is most nutritious and the health of cattle most vulnerable (the traditional, and current, disease avoidance strategy), or to stay and risk infection and disease. The potential impacts of the traditional disease avoidance strategy vary in different areas but include a) an increased disease burden from vector-borne and directly transmitted diseases due to the confinement of large numbers of cattle herds, often in woodland areas where vectors of disease are more concentrated, b) reduced access to salt, c) losses resulting from the energy demands associated with traveling large distances away from the wildebeest calving grounds and d) the impact of grazing poorer quality forage at a critical time of year for recovering body condition [2]. Consequently, the economic costs associated with the disease have been reported to be significant in regions where MCF risk to cattle is high [5–7]. These costs, however, have never been quantified.

Following recent advances in the development of a vaccine [8], field trials are currently underway to determine the efficacy of a new MCF immunisation strategy. For livestock owners the direct and indirect benefits of an effective vaccine could be significant: fewer cattle would die from MCF each year and, rather than having to move away, immunized cattle could continue to graze on the nutritious pastures of the wildebeest calving grounds at a time when the grass is at its most nutritious.

This vaccine trial provided an opportunity to evaluate the potential economic benefits that could accrue from of an effective vaccine, through (1) avoiding household resource and management costs incurred moving cattle away during the wildebeest calving season, and (2) being able to graze cattle on the higher quality pasture of the wildebeest calving ground. To assess the management and resource costs associated with moving, we used household survey data. To estimate the costs associated with changes in livestock body condition that result from the traditional MCF avoidance strategy we a) compared body condition of cattle moved away to substitute pastures (cattle owned by Maasai householders—the *control* herd) with cattle belonging to an ongoing MCF vaccine field trial that were allowed to graze alongside wildebeest on their calving ground pastures (vaccine trial animals—the *treatment* herd) and b) used a hedonic price regression (a statistical model that allows estimation of the marginal contribution of a good’s attributes to its market price [9–11]). We hypothesize that livestock condition and value will increase more (or decrease less) for cattle which are able to graze alongside wildebeest calves on higher quality forage, than for cattle that have been moved to substitute pastures in the traditional way. Based on the output of the hedonic price regression, we monetize these comparative physical impacts in terms of their effects on the estimated market price of animals that are able to remain on the calving area and those that are moved away during calving season.

## Results

We present results about i) the management costs associated with MCF avoidance, and ii) the impact of MCF avoidance on the market value of cattle. We discuss the results of these two analyses in turn.

### MCF avoidance—herd management and associated costs

**Figure 2:**
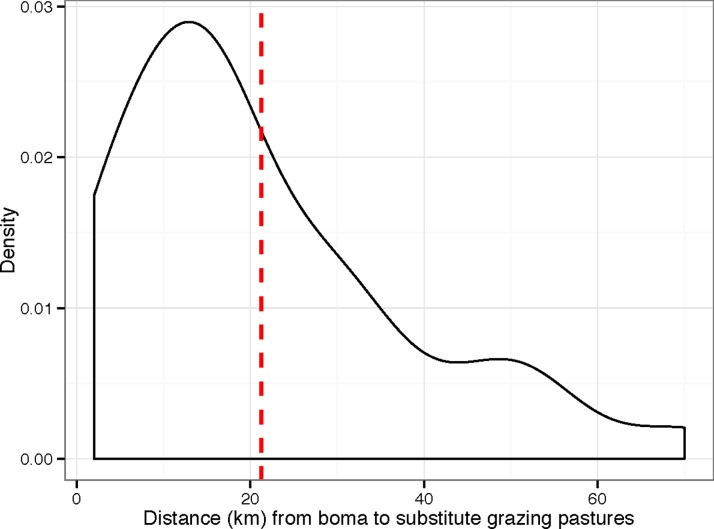
Density plot illustrating the distance (km) that cattle herds travelled to avoid wildebeest calves and MCF virus. Dashed red line indicates the mean

**Figure 3:**
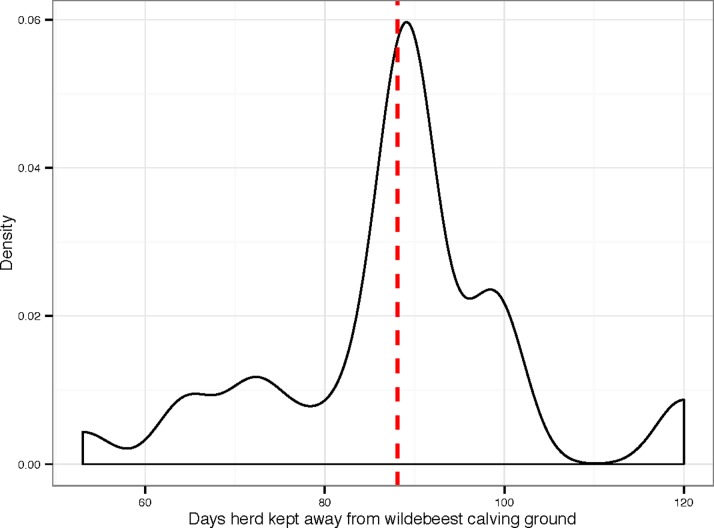
Density plot illustrating the length of time (days) cattle herds spend avoiding wildebeest calves and infection with the MCF virus. Dashed red line indicates the mean

**Figure 4:**
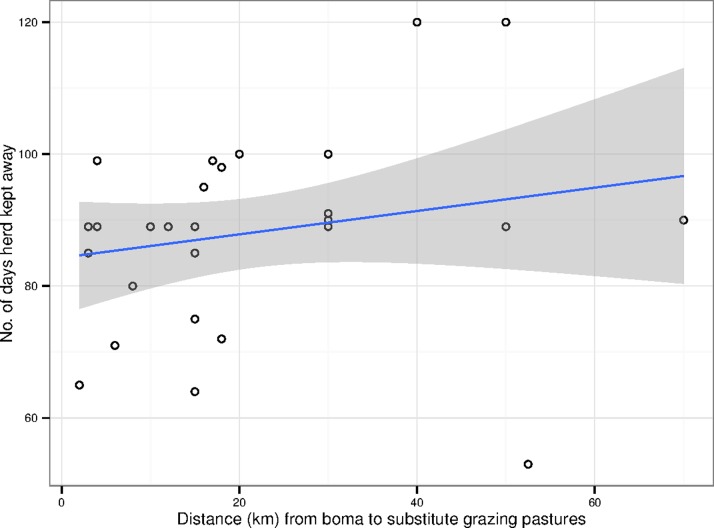
Scatter plot of the length of time (days) plotted against the distance travelled (km). The blue line and the grey areas indicate the regression line (*p*-value < 0.03, *r* = 0.4) and its confidence intervals respectively.

Table 1 provides an overview of the questions and summary statistics that emanated from the herd management questionnaire (the full data set is provided in S1 Table). Ninety percent of herd owners moved their cattle away from the wildebeest calving grounds to substitute pastures to avoid MCF during the calving season. The owners who did not move their cattle away had small herds consisting of less than seven head of cattle and they cited wanting to keep any milk produced at the boma (a traditional Maasai household unit) as the reason for not moving their herds. The substitute pastures to which cattle were moved were on average 21.3 km away from the boma (Fig. 2), took 2.2 days to reach and the cattle remained there for an average of 88 days (Fig. 3). The distance a herd was moved away from the wildebeest calving grounds to reach a substitute pasture area and the total time it spent away from the boma were moderately associated (*p*-value < 0.03, *r* = 0.4) (Fig. 4). Only 10% of herd owners employed non-family members to move and manage their cattle away from the boma, with 90% of households involving only members of the family unit. Within these households an average of 2.3 members (19% of the family unit) were recruited to move the cattle. Of the 90% of herds that moved away to avoid MCF, an average of 82% of each herd was moved, whilst 18% (an average of 15 cattle) were kept at the boma. Of the 18% of cattle that remained at the boma, an average of 74% were lactating. Despite lactating cattle being kept disproportionately at the boma and therefore at greater risk of MCF, 61% of all lactating cattle were moved to substitute pastures, resulting in an average of 71% of the total daily milk being produced by cattle that had been moved away. Of this milk only 10% was returned to the boma. Therefore, on average, 64% of the total daily milk was not available to be used by the 81% of the family unit that remained at the boma. The respondents reported that all of this milk was drunk by the herders or the calves, with none being sold or discarded. This constraint on milk allocation between herders and boma imposes costs on the household. To estimate an upper bound on this cost, we use a study of the Maasai community of Kitengela (Kenya), which found that 52% of the average gross annual household income is derived from milk, of which 27% comes from sales and 25% from consumption [12]. Assume briefly that none of the milk from the moved cows is consumed or sold, the loss of 64% of the household’s milk, which, if the Kitengela population is comparable, amounts to a loss of 33% of the household’s income during the calving season. Assuming that the cattle are away for 88 days (or 24% of the year), this upper-bound represents a loss of about 8% of the household’s annual income. Because the herders and calves do consume the milk the actual loss of income will be less than this. The calculation is useful, however, as it provides a limit above which the economic loss is unlikely to be.

**Table 1:** MCF management response.

				95% CI	
Variable	Results	SE	N	LL	UL
Prop. of herds that were moved away to avoid MCF	0.90	0.05	31	0.79	1.01
Prop. of each herd that moved away	0.82	0.07	28	0.67	0.96
Mean no. of cattle that remained at the boma	14.76	2.72	25	9.43	20.1
Prop. of lactating cattle moved away	0.61	0.02	503	0.57	0.65
Prop. of cattle not moved away that were lactating	0.74	0.12	14	0.51	0.97
Prop. daily milk derived from cattle away from boma	0.71	0.02	792	0.68	0.74
Prop. of milk returned to family at boma	0.10	0.08	15	-0.05	0.25
Mean time herd spent away (days)	88	2.60	30	82.97	93.16
Mean distance herd moved away (km)	21.3	3.10	30	15.20	27.37
Mean journey time to substitute grazing area (days)	2.2	0.38	30	1.43	2.91
Reason for moving cattle? *Avoid wildebeest*	0.94	0.04	30	0.86	1.02
Mean no. of family members moved with herd	2.27	0.21	30	1.85	2.69
Prop. family members moved with herd	0.19	0.10	16	0.00	0.38
Prop. herds that used non-family to move cattle	0.10	0.05	30	-0.01	0.21
Perception of wildebeest calving ground pasture: *Good*	0.52	0.09	31	0.34	0.70
Perception of wildebeest calving ground pasture: *Med*	0.48	0.09	31	0.30	0.66
Perception of substitute grazing pasture: *Good*	0.42	0.09	31	0.25	0.59
Perception of substitute grazing pasture: *Med*	0.55	0.09	31	0.37	0.73
Perception of substitute grazing pasture: *Low*	0.03	0.03	31	-0.03	0.09
Practise differ if MCF not a problem? *Yes*	0.90	0.05	31	0.79	1.01
How would practise differ? *Would not move*	0.96	0.04	28	0.89	1.03
How would practise differ? *Treat less for tryps*	0.11	0.06	28	-0.01	0.23
How would practise differ? *Cultivate more*	0.04	0.04	28	-0.03	0.11
How would practise differ? *Dip less*	0.07	0.05	28	-0.02	0.16

This table contains an overview of the questions and summary statistics that emanated from the herd management questionnaire

When asked for reasons why the herders had chosen to move their cattle away from the wildebeest calving grounds (and their village pasturelands), 94% said that they had moved away to avoid wildebeest, whilst 6% said they moved their cattle to seek better pasture. The quality of pasture, as perceived by the respondents, at the substitute grazing areas (high 42%, medium 55%, and low 3%) was lower than that of the wildebeest calving grounds (high 52%, medium 48% and low 0%). If MCF were no longer a health risk for their cattle 90% of herd owners say they would change their management practises, with 96% stating that they would no longer move their cattle away from the wildebeest calving grounds.

### MCF avoidance—health and condition associated costs and the impact on value

We now present the hedonic price regression results, a comparative summary of the impacts of body condition score (*BCS*) and heart girth (*HG*) on price, and a comparison of the changes in cattle condition and market value between the treatment and control herds. Table 2 provides a description of the attribute data retained in the hedonic price regression (the full set of questions and data collected by the market sample is given in S2 Table), Table 3 provides summary statistics and price effects for all these variables, whilst Table 4 contains the final hedonic regression results.

**Table 2:** Data description.

Data variable	Description
Age	Age of cattle in months
BCS	Body condition score (1 (thin)—5 (fat))
HG	Heart girth (cm)
Male	Male = 1, female = 0
Heifer	Female before first calving = 1; otherwise = 0
Time point 1	6th February 2012
Time point 2	4 March 2012
Time point 3	1st April 2012
Time point 4	24th November 2012

This table contains a description of the retained attribute data collected from cattle sold at livestock markets and from the control and treatment herds.

**Table 3:** Summary statistics for market sample, treatment and control herds.

herd	variable	mean	sd	min	max	N
Market sample	*Price*	235.2	88.7	100	575	185
	*HG*	124.7	15	90	162	185
	*BCS*	2.8	0.5	1	4	185
	*Age*	35.8	23.5	11	120	185
	*Male*	0.6	0.5	0	1	185
	*Heifer*	0.2	0.4	0	1	185
Control herd	*HG*	132.8	19.5	63	180	836
	*BCS*	3.1	0.5	1	5	839
	*Age*	42.4	26.6	6	166	1000
	*Male*	0.4	0.5	0	1	1000
	*Heifer*	0.4	0.5	0	1	996
Treatment herd	*HG*	124.9	9.1	100	154	393
	*BCS*	2.9	0.4	2	4	393
	*Age*	17.4	5.4	6	40	400
	*Male*	0.7	0.4	0	1	400
	*Heifer*	0.3	0.4	0	1	400

This table provides summary statistics for all variables used in the final regression and price effect estimation

**Table 4:** Hedonic price regression.

Variable	Estimate	t-stat
ln(*HG*)	-26.464	3.81^***^
ln(*HG*)^2^	2.901	3.98^***^
ln(*HG*)×ln(*BCS*)	0.241	5.81^***^
ln(*BCS*)^2^	-0.483	3.95^***^
ln(*Age*)	-2.920	1.54
ln(*Age*)^2^	0.875	1.56
ln(*Age*)^3^	-0.081	1.51
*Male*	0.090	1.23
*Heifer*	0.202	2.68^***^
I[Feb]	67.649	4.12^***^
I[Mar]	0.073	1.77^*^
I[Apr]	0.218	4.57^***^
I[Nov]	0.178	4.27^***^
Constant		
R-sq	0.78	
*N*	185	

*p < 0.1;

**p < 0.05;

***p < 0.01 (The heteroskedasticity-robust Huber/White/sandwich covariance estimator was used to calculate standard errors)

Joint significance of *Age*: $F_{171}^{3}=4.5$, *p* = 0.0047.

This table contains the results of the final hedonic price regression

Only cattle characteristics that were immediately verifiable by the buyer had a significant impact on price and were retained in the final regression. Other unverifiable characteristics, including whether or not the individual had been immunised against the locally prevalent disease east coast fever (ECF), the reported daily milk yield or number of previous calves, had no effect. The results show that, with all else constant, the market value of an animal begins to decline at about 86 months (7.2 years) and that the characteristics *HG*, *BCS*, *Age*, *Male* and *Heifer* are statistically important determinants of market value. Of these variables, only *HG* and *BCS* can be impacted by husbandry conditions and are of interest to the discussion regarding impacts of disease avoidance on value. Table 5 provides the elasticities of market price with respect to *BCS*, *HG*, and *Age*, evaluated at the means of the market data. Both *HG* and *BCS* have positive effects on price, but the effect of *HG* on price is ten times larger than that of *BCS*, and is more strongly significantly different from zero.

**Table 5:** Table of marginal effects.

Variable	Estimate	z-stat	*p*-value
ln(*BCS*)	0.177	1.62	0.105
ln(*HG*)	1.742	9.47	<0.001
ln(*Age*)	0.133	3.39	0.001

This table contains the marginal effects of *BCS, HG*, and *Age*, evaluated at sample means from the market dataset

The percentage change in the mean values of *HG* and *BCS* and mean price P^*^ that occurred in time periods 1, 2 and 3 are recorded for each herd in Table 6 (the full set of condition data is shown in S3 Table). Below we present the results including only cattle that made it through to the end of the study.

**Table 6:** Means test by herd and time period for treatment and control herds.

Time period	Name	Control	Treatment	t-stat	df^*^	*p*-value
1	% change in *BCS*	−5.4	0.8	−3.4	140	0.0010
1	% change *HG*	0	3.3	−3.4	180	0.0009
1	% change in ${\hat{P}}_{i}^{*}$	−0.9	5.0	−3.2	215	0.0015
2	% change in *BCS*	11.9	4.2	4.2	196	<0.0001
2	% change in *HG*	3.4	6.5	−4.0	219	0.0001
2	% change in ${\hat{P}}_{i}^{*}$	6.8	13.4	−3.9	221	0.0001
3	% change in *BCS*	−11.8	−23.8	4.3	220	<0.0001
3	% change *HG*	−1.3	−8.6	10.2	223	<0.0001
3	% change in ${\hat{P}}_{i}^{*}$	−6.4	−22.2	9.2	221	<0.0001

*Satterthwaite’s approximate degrees of freedom given unequal variances.

The table contains the herd specific percentage change in mean *HG, BCS* and net price P* that occurred in time periods 1, 2 and 3

**Time period 1**: In this period, during which the treatment herd remained on the pastures of the wildebeest calving grounds whilst the control herd was moved approximately 50 kilometres to substitute grazing areas, the change in the control herd mean *BCS* (− 5.4%) was significantly different (*p* = 0.001, *t* = −3.37, d.f = 140) from that of the treatment herd (+ 0.76%). The mean *HG* for the control herd remained constant, in comparison to the treatment herd which increased by 3.3% (*p* < 0.001, *t* = −3.39, d.f = 180). The combined effect of changes in *BCS* and *HG* are reflected in the differences in the change in mean price, P^*^, which, in time period 1, increased by 5% for the treatment herd compared to a 0.9% decrease for the control herd (*p* = 0.0015, *t* = −3.22, d.f = 215). (These sample means and the t-tests for $\bar{{\hat{P}}_{t}^{*}}$ do not account for the variance in the predictions themselves, so while the t-tests test for differences in average predicted prices, they do not account for the sampling variation in these individual predicted prices themselves.)

**Time period 2**: In this period, during which the control herd was grazed entirely on substitute pastures, the treatment herd’s mean *BCS* increased by 4.2% whilst the control herd’s mean *BCS* increased significantly more, by 11.9% (*p* < 0.0001, *t* = 4.24, df = 196). In the same interval, the control herd’s mean *HG* increased by 3.4% whilst the treatment herd’s mean *HG* increased significantly more, by 6.5% (*p* = 0.0001, *t* = −3.98, df = 219). The mean price of cattle for both herds increased in this period, however the price increase experienced by the treatment herd (13.5%) was more than twice that of the control herd (6.8%) (*p* = 0.0001, *t* = −3.86, d.f = 221).

**Time period 3**: In this dry season period, during which the control herd had returned from the distant pastures to graze alongside the treatment herd, the mean *BCS* of the control and treatment herd decreased by 11.7% and 23.8% respectively (*p* < 0.0001, *t* = 4.28, df = 220), whilst the *HG* decreased by 1.3% and 8.6% respectively (*p* < 0.0001, *t* = 10.23, df = 223). The mean price decreased for both herds, however the mean price of a cow in the treatment herd decreased much more, decreasing by 23.8% compared to the 11.7% drop for the control herd (*p* < 0.0001, *t* = 9.2, d.f = 221).

### Occurrence of disease

**Figure 5:**
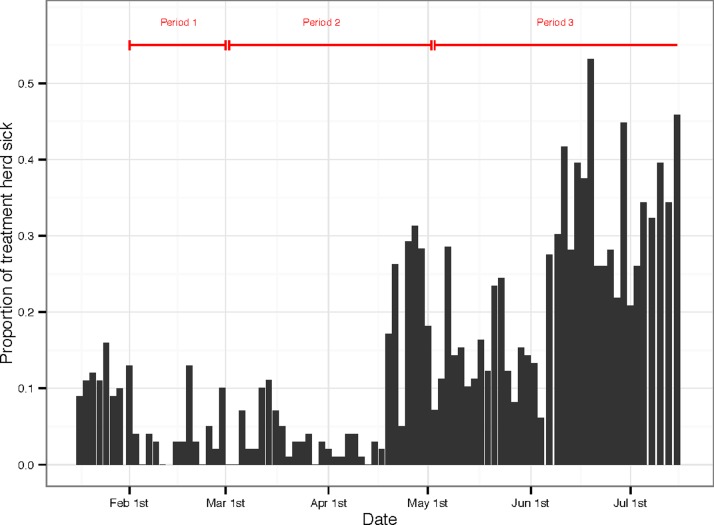
Bar chart of the proportion of the treatment herd cattle recorded sick during the study period. Data collection points were either 2 or 3 days apart and this accounts for the variation of the spacing of the bars. The plot is annotated with lines indicating the position of time periods 1–3.

During the entire study period 3% of the control herd cattle were reported to have had at least one days sickness, compared to over 90% of the treatment herd. Focussing only on the treatment herd: in time period 1 the mean daily percentage of the herd reported to be sick was 4.5%. By the end of time period 2, however, a respiratory infection had entered the herd and the daily mean percentage of the herd reported sick had increased to 7%, and in time period 3 (until the end of the vaccination trial in early July) the mean rose again to 24% (Fig. 5). The cause of the respiratory infection remains unknown, however analysis indicates that it was not MCF. This clinical data is relevant to the discussion that follows regarding the changes in body condition score and price.

## Discussion

This study, which builds on and supplements existing qualitative findings [1, 2], provides the first quantitative assessment of the annual costs that Maasai pastoralists incur as a result of the implementation of their traditional strategy to avoid the fatal livestock disease, wildebeest-associated malignant catarrhal fever. The economic costs assessed in this study can be categorized as (1) travel and other management costs and (2) livestock-productivity losses, which we discuss in turn.

In order to avoid contact with wildebeest and transmission of MCF most Maasai households moved their cattle away from the wildebeest calving ground during the infectious period, February to May. Not all cattle within a herd tended to be moved, however, with most owners choosing to keep a small number of cattle back at the boma. Approximately three quarters of the cattle that remained at the boma were lactating, reflecting the important role that milk plays both as a component of the diet and of the overall household income. As a consequence some of the most valuable animals faced the greatest risk of disease. In order to reduce this risk only a small number of animals, that could be grazed sufficiently on pastures near to the boma where the risk of exposure to wildebeest was lower, were kept by the family during the MCF season.

Despite this preference for keeping lactating cattle back at the boma nearly two-thirds of all lactating cattle were moved during MCF season, resulting in most of the total daily milk yield not being returned to be used by the majority of the family unit that remained at the boma. In traditional Maasai households, many of which survive on an energy intake of approximately 70% of the international recommended threshold [13, 14], most of the protein intake, and between between one third and a half of the energy intake, is derived from milk [14]. This reliance on milk has become more precarious recently with an expanding human population, a declining human / livestock ratio and increased sedenterization all decreasing the animal milk supply per person [15, 16]. Consequently the proportion of milk not returned to the boma during the three month MCF season is likely to represent a considerable seasonal nutritional loss to some households. Importantly, because herders are the young men this loss will predominantly affect the most economically vulnerable members of the household that remain at the boma, the women, the very young, and the elderly. Furthermore, the period that the cattle are moved to avoid MCF is the time of year when rainfall is at its peak and the fresh pasture at its most nutritious [17] and, as a consequence, milk supply usually at its highest [18]. Being unable to return 64% of the milk to the boma at this time of year will compromise a families capacity to convert the seasonal glut of milk into a form of preserved dairy product, such as cheese, or to sell it.

The flip side of this is that while the household members at the boma have less than they would have otherwise, the calves and herders receive a relative glut of milk to consume. Milk deflected to human consumption, though benefitting the pastoralist in the short-term, will depress the survival and the rate of maturation of young animals and therefore impair herd replacement and growth in the long-term [19]. Rather than being lost, therefore, the proportion of this glut of milk which is not returned to the family but is drunk by the calves themselves is literally being fed in to the household’s most secure long term investment. Such a scenario makes economic sense for better-off, calorifically sufficient, households with a relatively large number of livestock of which only 35–45% are typically milked. For poorer households, however, that have fewer livestock and reduced consumption options and that rely disproportionately on the livestock for milk (typically milking 65–75% of their herds) [20], investing the glut of milk in the health of their calves may be a constraint that imposes substantial costs in terms of current milk consumption for the family.

Milk has also been shown to play a significant role in the income stream of the average Maasai household [1, 12] and, using milk sales data from the Kitengela Maasai population, we have calculated that the loss of household milk during the calving season represents an upper bound of about 8% of the household’s annual income. Income elasticities for milk have been estimated to range around one for sub-Saharan Africa [21] and rural northern Tanzania in particular [22], which implies that for the upper bound milk production assumption, both milk consumption in the household, as well as consumption of all other goods (as an aggregate category) would each respectively decline by a similar percentage when the household are constrained from selling milk by it being produced far away from the boma and markets [23]. Despite the proximity to the town of Arusha, which has the highest rate of urbanisation of any region in Tanzania [24], it is likely that the percentage of household income derived from the sales of milk is lower for the rural Simanjiro Maasai population of this study than it is for the peri-urban Kitengela population that supplies the demands of Nairobi. Additionally, although none of the milk produced away from the boma is sold, none is discarded as it is either consumed by the herders or the calves, so these figures represent an upper bound on milk value loss. For these reasons we consider the upper bound estimate as higher than the actual loss experienced by the study population. The primary impact of MCF avoidance on milk usage, therefore, is through household distribution effects with the distance between the majority of milk production, the boma and point of potential sale representing an economic constraint that induces a reallocation of milk relative to how households, particularly the most impoverished, would choose to use the milk given the “unconstrained” case with zero MCF risk (and therefore no avoidance).

MCF avoidance induces further production losses through impacts on cattle body condition. We estimate these losses as reductions in market value, because market value represents the net value of the income stream from the cattle asset over its lifetime, and also represents the opportunity cost of holding (keeping) an animal rather than selling it.

In time period 1, the treatment herd, which grazed on the wildebeest calving ground, gained condition (both *BCS* and *HG*) whereas the control herd, moved to substitute grazing areas, lost condition (Table 6, row 1). Consequently the change in predicted value, directly attributable to these condition differences, increased by 6% more in the treatment than the control herd. The loss of body condition of the control herd is likely to be due to both the energy requirements of movement (with herds moved on average 23 km and maximally 88 km away from the wildebeest calving grounds), as well as the lower quality of pastures in the substitute areas where they grazed. While pasture quality was not measured directly, most of the Maasai in this study considered the wildebeest calving grounds to comprise the higher quality pasture. This is consistent with known drivers of wildebeest movement, with wildebeest in the Serengeti ecosystem known to move to high-quality pastures that are rich in nutrients and minerals during calving and lactation periods [25–27]. Should an effective vaccination allow all the local cattle herds to remain on the wildebeest calving ground then grazing competition might rise and the associated benefit of remaining on the pasture decrease. An ecological impact assessment carried out in the neighbouring Ngorongoro Conservation Area, however, predicted that forage availability was not a major factor in limiting cattle numbers [28] and as a consequence future pasture competition, following effective vaccination, may be relatively minor.

In time period 2, the *BCS* of the control herd rebounded, increasing by three times more than that of the treatment herd. Conversely, the treatment herd *HG* increased by almost twice as much as that of the control herd. Considering the different grazing strategies experienced by the two herds during this period, and the reported superior quality of pasture on the wildebeest calving ground, we expected that the changes in both *BCS* and *HG* would be greater for the treatment herd, but this was not the case. We hypothesize that this pattern of outcomes was due to two observed factors: (a) unforeseen supplemental feeding by the owners of the control herd which was reported after the end of the trial, and (b) the outbreak of a non-MCF respiratory infection that affected most of the treatment herd in time periods 2 and 3 (Fig. 5). During the same time period little or no illness was reported in the control herd. Thus the combination of supplemental feeding of the control herd and illness in the treatment herd may explain the result that *BCS* increased more in the control herd during this period. Despite the impact of this respiratory infection, the estimated change in value (% *change in* ${\hat{P}}_{i}^{*}$) of the treatment herd attributable to condition changes during this period increased by twice as much as that of the control herd, reflecting the fact that *HG* affects price by one order of magnitude more than *BCS* (Table 5).

During time period 3, which was also the dry season when livestock are expected to lose condition, the *BCS* and *HG* in both herds decreased, although the treatment herd lost more. This was likely the result of the high incidence of respiratory disease in this herd. As a consequence of these losses the portion of price attributable to condition decreased for both herds during the final time period although the loss in value experienced by the treatment herd was considerably higher than the control herd.

Given the disproportional incidence of the respiratory infection, it is difficult to estimate the full impact that traditional MCF avoidance strategies have on cattle value through changes in condition parameters. However, if we exclude time period 3, during which the sickness was most prevalent, and consider only periods 1 and 2 the increase in condition-associated value of the treatment herd (18.4%) was three times greater than that of the control herd (5.9%). We attribute this differential to the contrasting MCF disease avoidance strategies employed by the two herds. Given the dual impacts of the supplemental feeding of the control herd and the respiratory infection, which started to impact the treatment herd approximately three weeks before the end of time period 2, and that the calculation does not include any condition losses associated with the control herd returning to the wildebeest calving ground in May, we consider this differential conservative.

It has been reported that moving cattle to avoid MCF forces herds to graze in wooded areas, increasing exposure to insect vectors and associated diseases [1, 2]. This assertion is supported by some of the cattle owners in our sample who anticipate that, in the absence of MCF, they would spend less on medical treatment for trypanosomiasis and who predict that the frequency of dipping cattle would decrease. However in this study it was the treatment herd, grazing alongside wildebeest, that suffered more disease specifically because of the non-MCF respiratory infection that affected the herd. Not knowing the aetiology of the respiratory infection it is difficult to draw conclusions about its source however it is possible that the increased contact between the treatment herd and the wildebeest was a factor. If this is the case then the increased risk of non-MCF respiratory infection affecting MCF vaccinated herds needs to be included in future economic assessments of MCF vaccination.

Examination of the changes across the time points in Table 5 shows how fleeting *BCS* is, and it is therefore not surprising that differences in *BCS* have a smaller price effect than *HG*. If an animal is purchased thin or fat *BCS* can be altered by feeding or poor environment relatively rapidly. Whereas *HG*, which although affected in part by fattening, short-run nutrition and health fluctuations, primarily represents skeletal size and therefore productive capacity of an animal [29]. *HG* is therefore less fleeting, and this difference is reflected in the market price of the animal.

Finally, the labour and travel associated costs of MCF avoidance are also not insignificant. Most households send their cattle away to find substitute pastures to avoid the wildebeest and this requires several family members to spend on average three months away from home. Furthermore there are costs associated with the setting up and running of a second boma needed to house the herd and the travelling family members. Additionally, intermittent travel by foot between the primary and secondary boma has also been cited as an MCF associated cost [1]. Given the average distance moved (21.3 km), these ancillary travel costs are likely to be considerable.

## Conclusion

MCF presents the Maasai with an epidemiological and economic dilemma: Herd their cattle away from wildebeest calves to avoid disease, and incur costs from lost opportunities to consume and sell milk, and the energy and labour input required to move the cattle. Or, choose an alternative strategy to remain on the wildebeest pastures which, without an effective vaccine, also incurs costs through a higher risk of disease. Given the incidence of MCF in cattle living in wildebeest calving areas in East Africa is 5% to 10% [1, 30], the increase in disease costs associated with the alternative strategy will potentially offset any gains made through increased availability of milk, improved body condition and reduced energy demands from movements. This suggests that the traditional strategy is currently the least costly option. With the development of an effective vaccine, however, the alternative strategy might become optimal. Before this can be determined the aetiology and impact of non-MCF respiratory infections, that might result from increased contact with wildebeest following vaccination, would need to be understood. Other areas of study also include measuring the impact that a reduction in opportunities to consume milk during the MCF season might have on the health of women and young children and quantifying the burden of vector-borne and directly transmitted diseases associated with the confinement of large numbers of cattle herds on substitute grazing areas.

## Materials and Methods

The human subject research was conducted according to relevant international guidelines and was approved by the approval board committee of the Tanzanian Commission of Science and Technology (permit nos.2011-213-ER-2005-141 and 2012-318-ER-2005-141). Informed oral consent was given by all participants. Written consent was not given as many of the participants were not literate. To ensure that the participants were consenting, a well respected local elder was present for all interviews. Before proceeding the elder explained the purpose of the study to the participants and translated all questions in to the vernacular language. Only when the elder was satisfied that the participant had fully understood the implications of our study, and was happy to be interviewed, could we proceed. When oral consent had been given the name of the respondent was written onto the interview sheet and the elder checked a box that stated that the interviewee was consenting to be interviewed. This procedure for obtaining oral consent was approved by the ethics committee of the Tanzanian Commission of Science and Technology. The animal research, which was non-invasive and was conducted according to international guidelines, was specifically permitted by the ethics committee of the Tanzanian Wildlife Research Institute.

### Trial setting

The study took place, with the approval and co-operation of the community, on communally owned land (latitude −3.952239, longitude 36.47537) in a mixed-use buffer zone 25–40 km east of Tarangire National Park in a village called Emboreet in northern Tanzania’s Simanjiro District (Fig. 1). The buffer zone is inhabited by transhumant pastoralists and agro-pastoralist communities and is an important dispersal area for wildlife such as wildebeest (*Connochaetes taurinus*) and zebra (*Equus quagga*) that seasonally move out of the national park during the wet season (November to April) to calve. The migration of these herbivores is driven by seasonal water resources and by the high levels of nitrogen and phosphorus in the area’s vegetation that result from underlying volcanic soils and which are important for lactating females [26, 31, 32]. These high quality pastures are referred to in this study as the wildebeest calving grounds.

### MCF avoidance—herd management and associated costs

The first part of the economic impact assessment aimed to assess MCF avoidance practices and the associated costs incurred by livestock herd owners. A questionnaire survey targeted 16 Maasai livestock owners, selected because they lived close to the wildebeest calving ground and their herds grazed these pastures when wildebeest were not present (Fig. 1). The survey was carried out four times in 2012. The questionnaire, which was delivered in the Maasai language before being translated to English and was trialled with bilingual participants not involved with the trial, asked the respondents about specific MCF-related management decisions and the impact that these decisions had on their households. The data from this survey are summarized both graphically and in tabular form to provide a basis for a qualitative discussion of the impact of management activities on households, including the distributional impacts of MCF avoidance within the household.

### MCF avoidance—health and condition associated costs

The second part of the study aimed to estimate the impact of MCF avoidance on cattle market value by comparing the difference in the market value of animals that were moved to avoid MCF (Fig. 1) against cattle that were not moved. A four-step strategy is followed, involving sample identification, data collection, regression analysis, and market value inference.

1. **Sample identification:** We use data from three sources:
  (a) A trial herd (*n* = 100) from an ongoing MCF vaccine field trial (hereafter called the treatment herd) which, in contrast with traditional Maasai herding practices, was grazed on the wildebeest calving area during the period February—May. All other husbandry conditions were locally typical.
  (b) A control herd, comprised of two privately owned herds (*n* = 100 & 150) selected on the basis of their size (we wanted the total number of control cattle to be approximately 2.5 times the number of the treatment herd), location (based in the village of Emboreet), and representativeness (locally typical breed and husbandry conditions and, importantly, moved away from the wildebeest calving ground during MCF season).
  (c) A market sample (*n* = 185) which represents a set of cattle individually sold at a primary livestock market in the Simanjiro District. Selection was determined by owner willingness to participate. Animal attribute data, analogous to that recorded for the treatment and control herd, was recorded as described below. In addition, the sale price of each animal was collected. All cattle in (a), (b), and (c) were Tanzanian short-horn.
2. **Data collection:** At four seasonally relevant time points, a broad set of physical attribute data were collected for all cattle belonging to the treatment and control herds and the market sample. The recorded attribute data included heart girth (cm); wither height (cm); body condition score (1 (thin)—5 (fat)); colour; age (months); the number of pairs of incisors; the vaccination status (with respect to east coast fever (ECF), anthrax / black quarter, and lumpy skin disease); sex; if female, whether a heifer; the outcome of the cow’s last calving; if male, whether castrated. All animal attribute data was collected by the same enumerator and, based on preliminary analysis, only a selection of the variables were used in the final regression. The attribute data collection time points were as follows:
  - Time point 1: Early February when typically the rains have begun and fresh new grass has started to grow. This is the beginning of the period when cattle are moved away from the wildebeest calving ground to substitute grazing pastures and represents the start of the trial period and the base-line for this study;
  - Time point 2: Early March, just after the wildebeest calving season, marks the mid-point of the period in which cattle are kept away from the wildebeest calving ground pastures;
  - Time point 3: Early May, the risk period for MCF transmission has passed and cattle managed in a locally typical manner have just returned to graze on the wildebeest calving ground pastures;
  - Time point 4: Mid-November, represents the end of the long dry season.
  Collection of data at these specific points enabled changes in non-fixed attribute data to be quantified across three time periods:
  - Time period I (time point 1 to 2): The control herd were moved away from the wildebeest calving ground to substitute pastures; the end of this period marks the beginning of MCF risk.
  - Time period II (time point 2 to 3): The control herd were grazed on substitute pastures throughout this period, whilst the treatment herd remained on the wildebeest calving ground; this is the high risk period for MCF.
  - Time period III (time point 3 to 4): The control herd have returned to graze alongside the treatment herd on the wildebeest calving ground for the duration of the long dry season; this is the post-risk MCF period.
  Disease data were also collected: Control herd owners were asked which of their cattle had suffered from ill-health during the preceding time period (or preceding two months for the first time point). Being an experimental herd for which health data was already being recorded, disease data were compiled for the treatment herd every two or three days.
3. **Hedonic price estimation:** Based on the attribute and sale price data from the market sample we estimated the parameters of a hedonic price regression [9, 11].
4. **Market value inference for control and treatment herds:** The attribute data from the treatment and control herds were inserted into to the hedonic regression, which provided an estimate of the market value of each animal in each of the herds based on its physical condition [10]. From these estimates, the average differences in market value across herds and across time periods were calculated. Consequently we were able to infer the herd-level impact of having to move cattle away from the wildebeest calving ground.

The attrition rate of control herd cattle was substantial with only 141 out of 250 cattle making it through to time point 4, with most (65%) being lost to follow up after time point 3. Because we are focusing on changes in cattle condition over time, our results below, and in Table 6 on condition and value changes, utilized only those animals who remained in the herd across all three time periods (those who did not disappear from our sample prior to time point 4). However, attrition could in principle affect the outcome of our analysis if animals died or were sold or given away due (at least in part) to differences in condition, which is likely. To assess this we repeated the means tests with the data on lost cattle included in our calculations until lost. For example, if an animal was sold from the control herd in time period 2, we included the changes in its condition in the first time period. We found that the results were not different qualitatively than if all data for lost animals were excluded, and therefore the inferences do not change.

### MCF avoidance—impacts on value

The hedonic price regression relates the market sale price (*P*_*i*_) to a set of the animal’s attributes **X**_*i*_ for a set of cattle *i* = 1…*N* sold at market. Hedonic price theory provides little guidance on the specific functional form of the regression relationship, except that market prices for goods are positive, so consider first the general linear price function

$$g(P_{i})=f(X_{i};\;\beta )\;+\;\varepsilon_{i},$$ (1)

where *g*(⋅) and *f*(⋅) are transformation functions and ${\text{X}}_{i}\text{β}=\sum_{k=1}^{K}X_{ik}\beta_{k}$ is an index function, linear in *K* attributes and associated parameters *β*, including an intercept term. For example, if *g*(*P*_*i*_) = *P*_*i*_ and *f*(**X**_*i*_; *β*) = **X**_*i*_ *β*, equation 1 is a linear regression *P*_*i*_ = **X**_*i*_ *β* + *ɛ*_*i*_. Nonlinear Box-Cox formulations of *g*(⋅) and *f*(⋅) are possible [33–35], of which linear or log-linear functional forms are the most commonly applied special cases. Likelihood Ratio tests based on preliminary Box-Cox regressions reject the linear form and fail to reject using the log of price as the dependent variable (*p* = 0.60). We therefore apply a log-linear model of the form

$$\ln\;(P_{i})=X_{i}\beta \;+\;\varepsilon_{i},$$ (2)

where the continuous variables in **X** may in general be natural logarithms of the original variables (which applies in our specific case), and quadratic interaction terms as described below are included to allow for interaction effects of the explanatory variables. Ordinary Least Squares applied to this regression using the market data (after transforming *P* to ln(*P*)) provides consistent parameter estimates $\hat{\beta }$ assuming a correctly specified model.

A full range of explanatory variables (attribute data) were included in **X**_*i*_ *β* for the first iteration of the hedonic regression (S2 Table). A final hedonic regression specification was selected based on economic theory and step-wise deletions guided by a combination of F- and T- tests for significance, AIC and BIC values. The heteroskedasticity-robust Huber/White/sandwich covariance estimator was used to calculate standard errors. The most important modelling decisions in this regards are as follows:

First, livestock attributes observable at the market are most likely to impact price (unsubstantiated claims of high milk production or vaccination are likely to be discounted by buyers). This hypothesis was supported from initial regressions. We therefore focused on observable attributes: (i) Heart girth (*HG*), a summary statistic that correlates strongly with skeletal size, is a statistically important explanatory variable for explaining price variation [36, 37]; (ii) body condition score (*BCS*), a summary measure for the visual determination of nutritional status of an animal [38]; and (iii) *Age*, a potentially important characteristic because, even if all physical characteristics are perfectly controlled for, the temporal distribution of costs and benefits from owning an animal vary over its lifetime and benefits or costs accrued in the distant future tend to be discounted relative to those accrued in the near future [39]. Indicator variables for sex (*male* = 1 if male, zero otherwise) and whether a female is a heifer (*heifer* = 1 if a female has not calved, zero otherwise) were also included.

Aggregate market prices naturally vary over the course of the year, so we included date-specific indicator variables to account for aggregate market price fluctuations. Logarithmic transformations of the continuous variables and a parsimonious set of linear and quadratic terms minimized the AIC and BIC information criteria.

A description of the retained attribute data is given in Table 2. The final regression used for price estimation is:

$$\ln(P_{i})\;=\;\beta_{0}\;+\;\beta_{1}\ln{\left(HG\right)}_{i}\;+\;\beta_{2}\ln{\left(HG\right)}_{i}^{2}\;+\;\beta_{3}\ln{\left(HG\right)}_{i}\ln{\left(BCS\right)}_{i}\;+\;\beta_{4}\ln{\left(BCS\right)}_{i}^{2}\;+\;\beta_{5}\ln{\left(Age\right)}_{i}\;+\;\beta_{6}\ln{\left(Age\right)}_{i}^{2}\;+\;\beta_{7}\ln{\left(Age\right)}_{i}^{3}\;+\;\beta_{8}I[Male_{i}]\;+\;\beta_{9}I[heifer_{i}]\;+\;\beta_{10}I[Feb]\;+\;\beta_{11}I[Mar]\;+\;\beta_{12}I[Apr]\;+\;\beta_{13}I[Nov].$$ (3)

A cubic polynomial for age was included to best allow for hypothesized curvature in price due to age, because lower-order polynomials alone did not conform to theory, and because the visual relationships between age and market price indicated a sinusoidal relationship. The three age-associated coefficients are jointly significant (F(3,171) = 4.49, *p*-value = 0.0047).

This final hedonic regression allows calculation of an estimated market price for any given animal on any given day. To do this we simply enter the cattle specific attribute values recorded for the treatment and control herds into Equation 3.

Our principle objective, however, is to quantify the impact on price that is caused by a change in those specific attributes that could potentially be affected by disease avoidance strategies. Consequently, from the list of attributes that made it through to the final hedonic regression, the attributes of interest to our analysis are *BCS* and *HG*.

The percentage change in price with respect to a percentage change in *BCS* (also known as the *elasticity* of price with respect to BCS) is

$$\begin{array}{c} \frac{\partial \ln(P)}{\partial \ln(BCS)}\;=\;\beta_{3}\ln(HG)\;+\;2\beta_{4}\ln(BCS)\\ \approx \frac{\%\Delta P}{\%\Delta BCS}. \end{array}$$ (4)

where %Δ*X* = (*X*_*j*_−*X*_*i*_)/*X*_*i*_ for a change of any variable from *X*_*i*_ to *X*_*j*_, and $\frac{\%\Delta P}{\%\Delta \text{BCS}}$ represents the percent difference in *P* with respect to a percent difference in *BCS* holding all else constant. The relationship between $\frac{\partial \ln(P)}{\partial \ln(\text{BCS})}$ and $\frac{\%\Delta P}{\%\Delta \text{BCS}}$ is asymptotically exact as changes in *BCS* approach zero, but is approximate for relatively larger changes in *BCS* (hence the use of ≈ in Equation 4). The coefficients on the indicator variables such as Male, Heifer and the date variables require careful interpretation as well. An unbiased estimator of the percentage change in the transformed dependent variable *P* with respect to a change in an indicator variable *I* from 0 to 1 is $\Delta \;\text{ln}\;P∕\Delta I=e^{\hat{\beta }-\hat{v[\hat{\beta }]}∕2}-1$, where $\hat{v}[\hat{\beta }]$ is the estimated variance of $\hat{\beta }$ , calculated using the Delta Method [40–42].

An estimate of percent change in price due to a percentage change in *BCS* from one time point, *t* to the next *t* + 1 (holding all else constant) is derived by multiplying both sides of Equation 4 by %Δ*BCS*:

$$\%\Delta_{t}P\approx \left(\frac{\partial \ln(P)}{\partial \ln(BCS)}\right)\%\Delta_{t}BCS,$$ (5)

where $\frac{\partial \ln(P)}{\partial \ln(\text{BCS})}$ is defined in Equation 4. Analogous elasticities for *HG* and *Age* can be derived (as first derivatives) from equation 3.

Equation 5 amounts to a difference equation with respect to ln(*HG*), ln(*BCS*), and ln(*Age*), and is useful for estimating the effect of *BCS* on price holding all else constant (the same holds for analogous difference equations for *HG* and *Age*). Note that *Male* and *Heifer* do not vary over time and so drop out, and that the date indicators, which were necessary only to control for market conditions for estimation, are set to zero for the following analysis. Consequently the estimated market price correspond to the base case which is the 1^*st*^ market sample time point.

As all three variables vary simultaneously, the difference in estimated ln(*P*) from one time point to the next, due to simultaneous changes in ln(*Age*), ln(*HG*), and ln(*BCS*), is:

$$\Delta_{t}\ln({\hat{P}}_{i})\;=\;{\hat{\beta }}_{1}\Delta_{t}\ln{\left(HG\right)}_{i}\;+\;{\hat{\beta }}_{2}\Delta_{t}\ln{\left(HG\right)}_{i}^{2}\;+\;{\hat{\beta }}_{3}(\Delta_{t}\ln{\left(HG\right)}_{i}\ln{\left(BCS\right)}_{i})\;+\;{\hat{\beta }}_{4}\Delta_{t}\ln{\left(BCS\right)}_{i}^{2}\;+\;{\hat{\beta }}_{5}\Delta_{t}\ln{\left(Age\right)}_{i}\;+\;{\hat{\beta }}_{6}\Delta_{t}\ln{\left(Age\right)}_{i}^{2}\;+\;{\hat{\beta }}_{7}\Delta_{t}\ln{\left(Age\right)}_{i}^{3}.$$ (6)

Were we to apply equation 6 to estimate the change in price over time for an animal it would include not only the effects of changes in *HG* and *BCS* but also the effects of ageing on price. As animals simultaneously age and change body composition over time the effects of *Age* should be netted out of the price changes to avoid conflating age and condition effects (*HG* is strongly correlated with total body weight, which tends to increase more for younger cattle in a given environment [29]). As the age structure of the treatment and control herds were very different (the treatment herd was selected for a vaccination trial, while the control herds were maintained for the benefit of households), netting out the affect of differences in age on price is important for isolating the effects of *HG* and *BCS*. To do this we subtracted the age effects from both sides, which provides:

$$\Delta_{t}\ln({\hat{P}}_{i}^{*})\;=\;{\hat{\beta }}_{1}\Delta_{t}\ln{\left(HG\right)}_{i}\;+\;{\hat{\beta }}_{2}\Delta_{t}\ln{\left(HG\right)}_{i}^{2}\;+\;{\hat{\beta }}_{3}\Delta_{t}(\ln{\left(HG\right)}_{i}\ln{\left(BCS\right)}_{i})\;+\;{\hat{\beta }}_{4}\Delta_{t}\ln{\left(BCS\right)}_{i}^{2},$$ (7)

where $\Delta_{t}\ln(P_{i}^{*})=\Delta_{t}\ln({\hat{P}}_{i})-\left({\hat{\beta }}_{5}\Delta_{t}\ln{\left(Age\right)}_{i}+{\hat{\beta }}_{6}\Delta_{t}\ln{\left(Age\right)}_{i}^{2}+{\hat{\beta }}_{7}\Delta_{t}\ln{\left(Age\right)}_{i}^{3}\right)$ is the estimated percent change in expected price due solely to changes in *HG* and *BCS*. The right hand side of equation 7 amounts to the difference equation for ln(*P*) with respect to changes in ln(*HG*) and ln(*BCS*) holding *Age* constant.

As described above, for any variable *X*, log-differences are approximately equal to percent changes: Δ_*t*_ln(*X*)_*i*_ ≈ %Δ_*t*_ *X*_*i*_ (the approximation is asymptotically exact as *X*_*i*, *t*_ − *X*_*i*, *t*−1_ becomes small). Therefore, including the percent changes %Δ_*t*_ *HG* and %Δ_*t*_ *BCS* across time points *t* = (*t* = 1→2, 2→3, 3→4) for each animal *i* in equation 7 along with the estimated coefficients provides an estimated percent change in price ($\%\Delta_{t}\ln({\hat{P}}_{i}^{*})$) due to changes in cattle condition. Comparison of the sample means ($\bar{\%\Delta_{t}{\hat{P}}_{i;h}^{*}}=(1/n_{h})\sum_{i=1}^{n_{h}}\Delta_{t}\;\text{ln}({\hat{P}}_{i;h}^{*})$) for each herd *h* = (*control, treatment*), where *n*_*h*_ is the number of animals in herd *h*, allows a herd-level comparison of changes in price due to changes in the condition of animals in each of the two herds.

### Approval

This study was approved by both the Tanzanian Wildlife Research Institute (TAWIRI) and the Commission for Science and Technology (COSTECH, Tanzania). The human subject research was conducted according to relevant international guidelines and was approved by COSTECH (permit nos.2011-213-ER-2005-141 and 2012-318-ER-2005-141). The animal research, which was non-invasive and was conducted according to international guidelines, was permitted by the Tanzanian Wildlife Research Institute (TAWIRI).

## Supporting Information

S1 Table Herd management data. The data was collected from 16 Maasai herd owners at each of the four time points (1–4).
(CSV)

S2 Table Cattle market data. Animal attribute condition data, analogous to that recorded for the treatment and control herd, was recorded. In addition, the sale price of each animal was collected and other variables that might have affected price.
(CSV)

S3 Table Cattle condition data. Body condition score (BCS) and heart girth (cm) recorded for the treatment and control herds at each of the time points (1–4).
(CSV)
