# Correction: The Economic Impact of Malignant Catarrhal Fever on Pastoralist Livelihoods

**DOI:** 10.1371/journal.pone.0223347

**Published:** 2019-09-26

**Authors:** Felix Lankester, Ahmed Lugelo, Rudovick Kazwala, Julius Keyyu, Sarah Cleaveland, Jonathan Yoder

## Notice of republication

An incorrect version of S1 Table was published in error. This article was republished on September 19, 2019 to correct for this error. Please download this article again to view the correct version.
